# Evaluation of Cell Type Annotation R Packages on Single-cell RNA-seq Data

**DOI:** 10.1016/j.gpb.2020.07.004

**Published:** 2020-12-24

**Authors:** Qianhui Huang, Yu Liu, Yuheng Du, Lana X. Garmire

**Affiliations:** 1 Department of Biostatistics, University of Michigan, Ann Arbor, MI 48109, USA; 2 Department of Computational Medicine and Bioinformatics, University of Michigan, Ann Arbor, MI 48105, USA

**Keywords:** scRNA-seq, Cell type, Annotation, Classification, Benchmark

## Abstract

Annotating **cell types** is a critical step in single-cell RNA sequencing (**scRNA-seq**) data analysis. Some supervised or semi-supervised **classification** methods have recently emerged to enable automated cell type identification. However, comprehensive evaluations of these methods are lacking. Moreover, it is not clear whether some classification methods originally designed for analyzing other bulk omics data are adaptable to scRNA-seq analysis. In this study, we evaluated ten cell type **annotation** methods publicly available as R packages. Eight of them are popular methods developed specifically for single-cell research, including Seurat, scmap, SingleR, CHETAH, SingleCellNet, scID, Garnett, and SCINA. The other two methods were repurposed from deconvoluting DNA methylation data, *i.e.*, linear constrained projection (CP) and robust partial correlations (RPC). We conducted systematic comparisons on a wide variety of public scRNA-seq datasets as well as simulation data. We assessed the accuracy through intra-dataset and inter-dataset predictions; the robustness over practical challenges such as gene filtering, high similarity among cell types, and increased cell type classes; as well as the detection of rare and unknown cell types. Overall, methods such as Seurat, SingleR, CP, RPC, and SingleCellNet performed well, with Seurat being the best at annotating major cell types. Additionally, Seurat, SingleR, CP, and RPC were more robust against downsampling. However, Seurat did have a major drawback at predicting rare cell populations, and it was suboptimal at differentiating cell types highly similar to each other, compared to SingleR and RPC. All the code and data are available from https://github.com/qianhuiSenn/scRNA_cell_deconv_benchmark.

## Introduction

Single-cell RNA sequencing (scRNA-seq) has emerged as a powerful tool to enable the characterization of cell types and states in complex tissues and organisms at the single-cell level [1], [2], [3], [4], [5]. Annotating cell types amongst cell clusters is a critical step before other downstream analyses, such as differential gene expression and pseudo time analysis [6], [7], [8], [9]. Conventionally, a set of previously known cell type-specific markers is used to label clusters manually. This process is laborious and often a rate-limiting step for scRNA-seq analysis. It is also prone to bias and errors. The marker may not be specific enough to differentiate the cell subpopulations in the same dataset or be generic enough to be applied from one study to another. Automating cell type labeling is critical for enhancing reproducibility and consistency among single-cell studies.

Recently some annotation methods have emerged to systematically assign cell types in a new scRNA-seq dataset based on existing annotations from another dataset. Instead of using only top differentiating markers, most methods project or correlate new cells onto similar cells in the well-annotated reference dataset, by leveraging the whole transcriptome profile. These annotation methods have developed rapidly. However, benchmark datasets that the bioinformatics community agrees upon are lacking. These issues pose an urgent need to comprehensively evaluate the annotation methods using datasets with different biological variabilities, protocols, and platforms. It is essential to provide practical guidelines for users. Identification of each method’s advantages and limitations will provide practical guidelines and help boost further algorithmic development, which in turn will benefit the scRNA-seq community.

In this study, we evaluated ten cell annotation methods publicly available as R packages (Table 1). Eight are popular methods developed specifically for single-cell research (Seurat [10], scmap [11], SingleR [12], CHETAH [13], SingleCellNet [14], scID [15], Garnett [16], and SCINA [17]). Those methods can be further divided into two categories: Seurat, scmap, SingleR, CHETAH, SingleCellNet, and scID utilize the gene expression profile as a reference without prior knowledge in signature sets, while Garnett and SCINA require additional pre-defined gene markers as inputs. To leverage existing deconvolution methods for bulk omics data, we also included two modified methods: linear constrained projection (CP) and robust partial correlations (RPC) that are popular in DNA methylation analysis [18]. We conducted systematic comparisons on six publicly available scRNA-seq datasets (Table 2), varied by species, tissue types, and sequencing protocols, as well as five sets of simulation data with known true measurements.

**Table 1:** **List of scRNA-seq and methylation cell type annotation tools benchmarked in this study**

**Software**	**Method/algorithm**	**Bulk/single-cell reference data**	**Pre-defined marker genes required**	**Unknown cell types allowed**	**Software version in R 3.6.0**	**Ref.**
SingleR	Correlation-based with iterative tuning	Bulk	No	No	SingleR_1.0.0	[12]
CP	Reference-based method using CP	Bulk	No	No	EpiDISH_2.0.2	[18]
RPC	Reference-based RPC	Bulk	No	No	EpiDISH_2.0.2	[18]
Garnett	Elastic net multinomial regression	Single-cell	Yes	Yes	garnett_0.1.4	[16]
SCINA	Bimodal distribution assumption for marker genes	Single-cell	Yes	Yes	SCINA_1.1.0	[17]
Seurat	Define anchor with CCA, L2-norm, and MNN	Single-cell	No	No	Seurat_3.0.1	[10]
SingleCellNet	Multi-class random forest	Single-cell	No	No	SingleCellNet_0.1.0	[14]
CHETAH	Correlation-based with hierarchical classification	Single-cell	No	Yes	CHETAH_1.1.2	[13]
scmap	KNN classification with cosine similarity	Single-cell	No	Yes	scmap_1.6.0	[11]
scID	Fisher's linear discriminant analysis-like framework	Single-cell	No	Yes	scID_0.0.0.9000	[15]

*Note*: CP, constrained projection; RPC, robust partial correlation; CCA, canonical-correlation analysis; MNN, mutual nearest neighbors; KNN, K-nearest neighbors.

**Table 2:** **Datasets used in this study**

**Dataset name**	**Protocol**	**No. of cells**	**No. of genes**	**No. of cell types**	**Species/tissue description**	**Refs.**
PBMC sorted	10X	91,649	18,986	7	Human PBMCs	[33]
PBMC-3K	10X	2467	13,714	6	Human PBMCs	
Pancreas sorted	CEL-Seq2	2285	34,363	13	Human pancreas	[10], [31]
Pancreas	Fluidigm C1	638	34,363	13	Human pancreas	[10], [32]
TM full sorted	Smart-Seq2	24,622	22,252	37	Mouse	[3]
TM full	10X	20,000	17,866	32	Mouse	[3]
TM lung sorted	Smart-Seq2	1563	22,253	10	Mouse lung	[3]
TM lung	10X	1303	17,866	8	Mouse lung	[3]
Simulation 1 true	Splatter	2000	4000	5	Simulation data for cross-dataset prediction	
Simulation 1 raw	Splatter	2000	4000	5	Simulation data for cross-dataset prediction	
Simulation 2 true	Splatter	2000	10,000	5	Simulation data with increasing differential expression scales from low, low–moderate, moderate to high, each generated with 5 random seeds	
Simulation 2 raw	Splatter	2000	10,000	5	Simulation data with increasing differential expression scales from low, low–moderate, moderate to high, each generated with 5 random seeds	
Simulation 3 true	Splatter	10,000	20,000	10/20/30/40/50	Simulation data with increasing No. of cell type classes from 10 to 50	
Simulation 3 raw	Splatter	10,000	20,000	10/20/30/40/50	Simulation data with increasing No. of cell type classes from 10 to 50	
Simulation 4 true	Splatter	2000	10,000	9	Simulation data with descending cell proportion for each cell group, generated with 10 random seeds	
Simulation 4 raw	Splatter	2000	10,000	9	Simulation data with descending cell proportion for each cell group, generated with 10 random seeds	
Simulation 5 true	Splatter	5000/10,000/15,000/20,000/25,000/50,000	20,000	5	Simulation data with increasing No. of cells from 5000 to 50,000	
Simulation 5 raw	Splatter	5000/10,000/15,000/20,000/25,000/50,000	20,000	5	Simulation data with increasing No. of cells from 5000 to 50,000	

*Note* PBMC-3K data were obtained from https://support.10xgenomics.com/single-cell-gene-expression/datasets/. Raw data indicate the true simulation data with the addition of dropouts. Sorted data were generated from the fluorescence-activated cell sorting. TM, *Tabula Muris*; PBMC, peripheral blood mononuclear cell.

## Results

### Intra-dataset annotation and performance comparison

**Supplementary figure S1:**
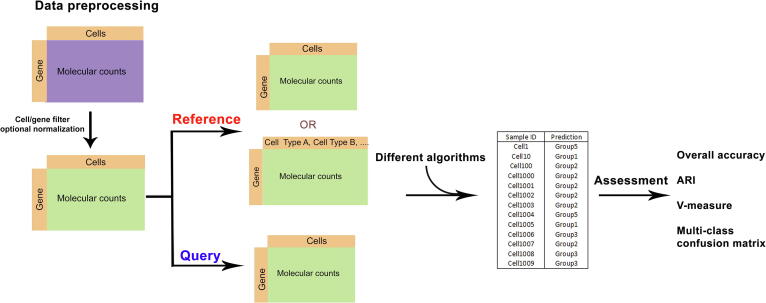
**Benchmark workflow**. The illustration of the workflow for this study consists of 1) preprocessing, 2) prediction, and 3) evaluations. ARI, adjusted rand index.

We first tested the classification accuracy of ten methods (Table 1) on six publicly available scRNA-seq datasets (Table 2). These datasets include two peripheral blood mononuclear cell (PBMC) datasets, two human pancreatic islet datasets, and two whole mouse datasets (*Tabula Muris*, or TM full). Since *Tabula Muris* datasets are heterogeneous in terms of tissue contents, to evaluate the tools’ performance on homogeneous data, we downsampled them separately into two mouse lung datasets (*Tabula Muris* lung, or TM lung) by taking cells from lung tissue. This resulted in eight real scRNA-seq datasets (Table 2). To avoid potential bias, we used the 5-fold cross validation scheme to measure the averaged accuracy in the 1-fold hold-out subset. We used three different performance measurement metrics: overall accuracy, adjusted rand index (ARI), and V-measure [19], [20] (see Materials and methods). The evaluation workflow is depicted in Figure S1.

**Figure 1:**
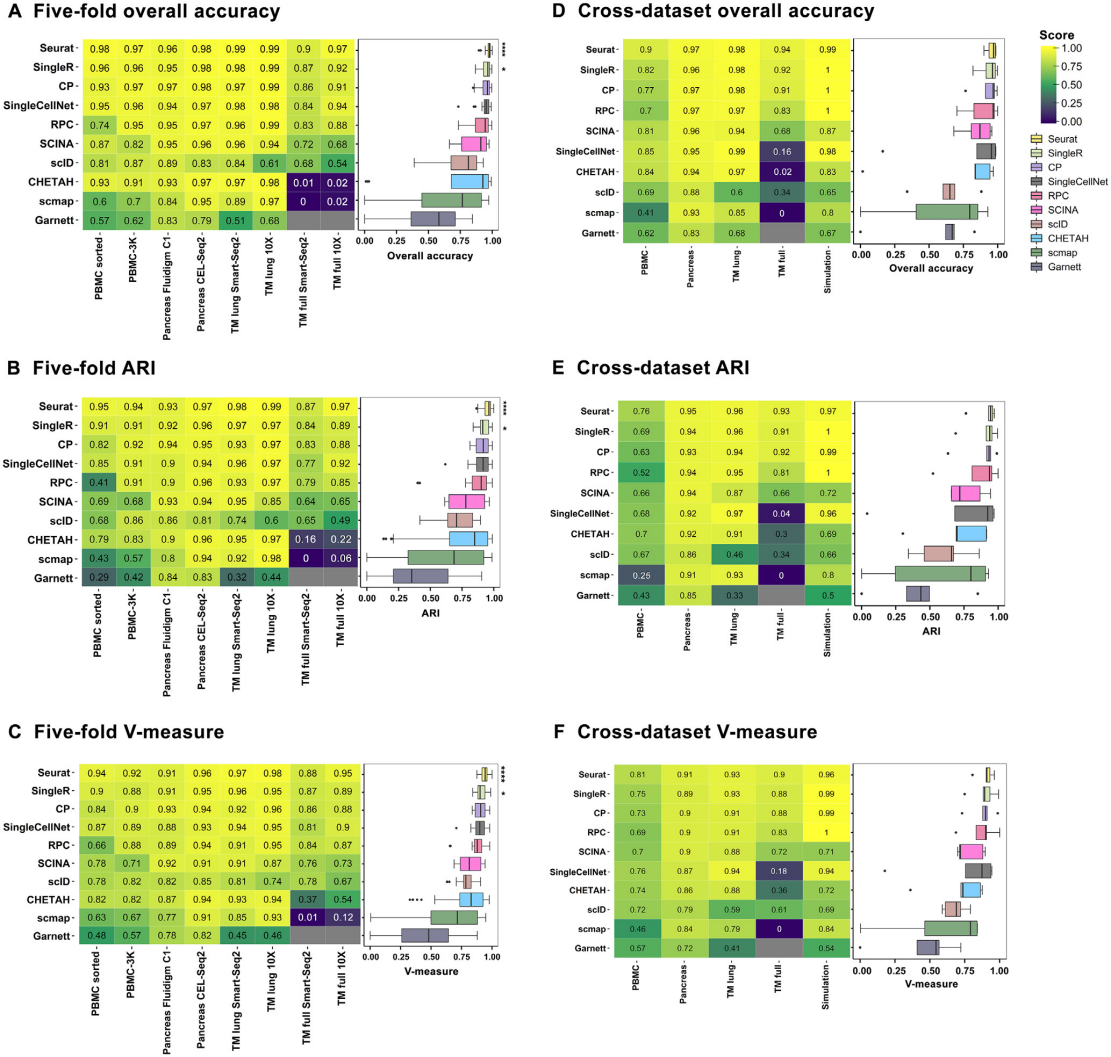
**Intra-dataset and inter-dataset accuracy comparison** **A.**–**C.** Results of intra-dataset accuracy comparison over eight real datasets are shown as heatmaps of three classification metrics: overall accuracy (A), ARI (B), and V-measure (C). For each dataset, a 5-fold cross validation was performed: using 4-fold as the reference and 1-fold as the query. **D.**–**F.** Results of inter-dataset accuracy comparison over four pairs of experimental datasets and one pair of simulation datasets are shown as heatmaps of three classification metrics: overall accuracy (D), ARI (E), and V-measure (F). PBMC pair: PBMC sorted and PBMC-3K; pancreas cell pair: pancreas CEL-Seq2 and pancreas Fluidigm C1; TM full pair: TM full Smart-Seq2 and TM full 10X; TM lung pair: TM lung Smart-Seq2 and TM lung 10X; simulation: true assay and raw assay. TM lung datasets were downsampled from TM full datasets by taking cells from lung tissue only. Within the simulation dataset pair, the true assay without dropouts was used as the reference and the raw assay with dropout mask was used as the query. The columns are datasets, and the rows are annotation methods. The heatmap scale is shown on the figure, where the brighter yellow color indicates a better classification performance. On the right of each heatmap is a boxplot to summarize the classification metrics across all datasets for each method. Box colors represent different methods. The methods in the heatmap and the boxplot are arranged in descending order by their average metrics across all datasets. Some methods failed to produce a prediction for certain datasets (indicated by gray squares). ^****^, significantly higher (*P* < 0.05) than nine other methods using Wilcoxon paired rank test; *, significantly higher (*P* < 0.05) than six other methods using Wilcoxon paired rank test. ARI, adjusted rand index; PBMC, peripheral blood mononuclear cell; TM, *Tabula Muris*.

Figure 1A–C show the classification performance on eight datasets. The five top-performing annotation methods were Seurat, SingleR, CP, SingleCellNet, and RPC. Seurat had the best overall classification performance in the 5-fold cross validation evaluation. On average, the three evaluation metrics from Seurat were significantly higher (Wilcoxon paired rank test, *P* < 0.05) than the other nine methods. SingleR had the second-best performance, with all three metrics higher than the other eight methods, among which six pair-wise method comparisons achieved statistical significance (Wilcoxon paired rank test, *P* < 0.05). Though slightly lower in average metrics, the classification performance of both SingleCellNet and CP was comparable to SingleR.

In order to test the influence of the cell type classes on the tools’ performance, we next evaluated the TM full and TM lung results. As shown in Table 2, for two TM full datasets from 10X and Smart-Seq2 platforms (with 32 and 37 cell types, respectively), we used a subset to create two smaller TM lung datasets (with 8 and 10 cell types, respectively). Most methods performed well for both TM lung datasets with ARI > 0.9. However, some methods did not have high performances on the two TM full datasets as the increased classification labels imposed challenges. Garnett failed to perform prediction on such large TM full datasets. Additionally, SCINA, CHETAH, and scmap had significantly lower classification metrics on TM full datasets than on TM lung datasets. In contrast, the previously mentioned top-five methods were more robust despite the increase of complexity in TM full datasets. Again, Seurat yielded the best performance in both TM full datasets, demonstrating its capability at analyzing complex datasets.

### Inter-dataset annotation and performance comparison

To evaluate the annotation tools in a more realistic setting, we conducted inter-dataset performance evaluation on ten datasets (five pairs), where the referencing labels were obtained from one dataset and the classification was done on the other dataset of the same tissue type (Table 2). Within a pair, we used the fluorescence-activated cell sorting (FACS) sorted dataset as the reference data, and the remaining one as the query data (see Materials and methods). Among the five pairs of datasets, four were real experimental data: PBMC pair with PBMC sorted data as the reference and PBMC-3K data as the query; human pancreas cell pair with pancreas CEL-Seq2 data as the reference and pancreas Fluidigm C1 data as the query; TM full pair with TM full Smart-Seq2 data as the reference and TM full 10X data as the query; TM lung pair with TM lung Smart-Seq2 data as the reference and TM lung 10X data as the query. The last pair was simulation datasets with the pre-defined truth, where the true assay without dropouts was used as the reference, and the raw assay with dropout mask was used as the query.

**Supplementary figure S2:**
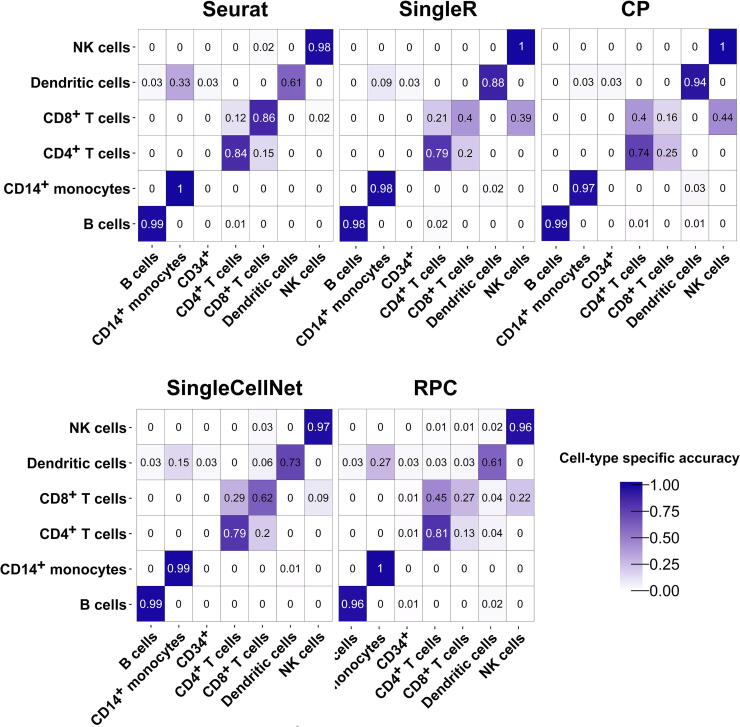
**Confusion matrix for top-5 performing methods on PBMC inter-dataset prediction**. Confusion matrix of cell-type-specific accuracy for PBMC inter-dataset predictions among top performing annotation methods (Seurat, SingleR, CP, SingleCellNet, and RPC). The *x*-axis is the predicted label from each algorithm, and the *y*-axis is the true label in the query data.

Figure 1D–F show the classification performance on the aforementioned five pairs of query and reference datasets. The top-three performing annotation methods in descending rank order were Seurat, SingleR, and CP, same as the intra-dataset cross validation results (Figure 1A–C). In particular, they all performed well on the simulation data with known true measurements, as the three metrics were all above 0.96. RPC was ranked 4th, slightly better than SCINA. Similar to the 5-fold cross validation evaluation, methods such as scID, CHETAH, scmap, and Garnett were consistently ranked among the lowest-performing methods for classification metrics. Interestingly, SingleCellNet, the method that performed relatively well (ranked 4th) in the intra-dataset cross validation, was now ranked 6th, behind RPC and SCINA, due to the drop of performance in TM full datasets. The accuracy scores were also much dependent on the datasets. For example, on complex PBMC datasets, even Seurat only reached 0.76 for ARI. A further examination of the confusion matrix (Figure S2) for Seurat, SingleR, CP, SingleCellNet, and RPC revealed that the challenge arose when distinguishing highly similar cell types such as CD4^+^ T cells *vs.* CD8^+^ T cells or dendritic cells *vs.* CD14^+^ monocytes in PBMC datasets.

**Supplementary figure S3:**
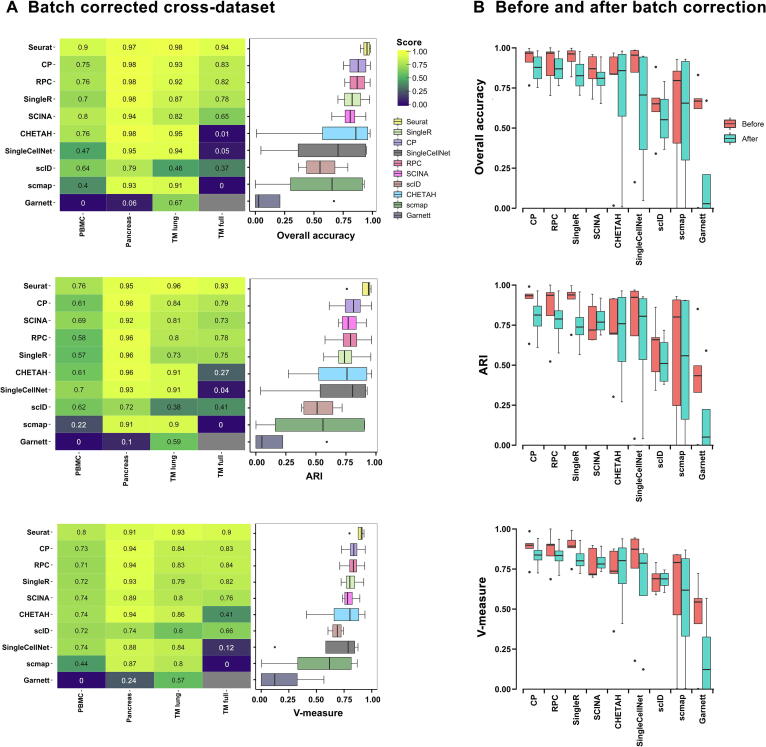
**Inter-dataset prediction using aligned reference and query matrix**. **A.** Inter-dataset accuracy comparison, shown as heatmaps of three classification metrics (overall accuracy, ARI, and V-measure). **B.** Boxplots illustrating the averaged metric scores before and after alignment for each method. The *x*-axis is the methods, and the *y*-axis is the classification metrics. For each of the four pairs of experimental data used in inter-dataset evaluation, we aligned both reference and query data using CCA from the Seurat data integration function. Then we separated aligned datasets and performed the inter-dataset evaluation again.

We also performed batch-corrected inter-dataset predictions on four pairs of experimental datasets. For each pair of data, both reference and query datasets were aligned using canonical-correlation analysis (CCA) [10], [21]. The result is illustrated in Figure S3A and B. Most methods did not benefit from aligning or integrating the datasets (Figure S3B). None of the other methods exceeded the performance of Seurat in any of the three metrics after batch correction (Figure S3A). The drop of performance in those methods may be attributed to the fact that aligned datasets contain negative values after the matrix correction and subtraction from the integration algorithm used in Seurat. In addition, some algorithms require non-normalized data matrix as the input, while batch-corrected matrix from Seurat is normalized. This may violate some models’ assumptions.

Altogether, these results from both experimental and simulation data demonstrate that Seurat has the best overall performance among the annotation methods, based on intra-dataset prediction and inter-dataset prediction.

### The effect of cell type similarity on performance

**Figure 2:**
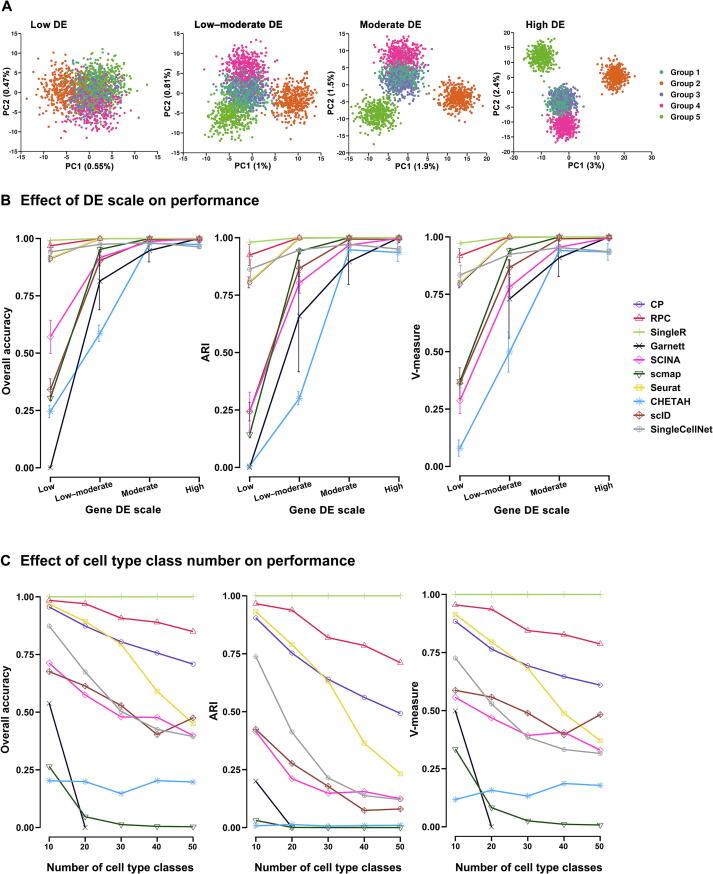
**Effect of increased DE scalesand cell type classes on annotation performance** **A.** PCA plots of simulation datasets generated by Splatter. Each dataset is composed of 10,000 genes and 2000 cells, splitting into 5 cell types with equal proportion. Each dataset contains the same proportion of DE genes in each cell type. The datasets differ by the magnitude of DE factors for those DE genes to modify cell–cell similarities. We generated 20 datasets with the cell group similarity ranging from low, low–moderate, moderate to high DE (see Materials and methods). Colors represent different cell types. **B.** The evaluation of each annotation method applied to the datasets in (A) is shown by plots of three classification metrics: overall accuracy, ARI, and V-measure. The *x*-axis is the gene DE scale in each cell group, and the *y*-axis is the metric score. Results are shown as mean ± SD over five repetitions. Line colors and point shapes correspond to different methods. **C.** The performance of each method for increased cell type classes is shown by plots of three classification metrics: overall accuracy, ARI, and V-measure. Each simulation dataset is composed of an increased number (*N*) of cell types (*N* = 10, 20, 30, 40, 50) with constant total cell number (10,000), gene number (20,000), and DE level among cell types. The *x*-axis is the number of cell type classes in each dataset, and the *y*-axis is the metric score. PCA, principal component analysis; DE, differential expression.

Since it is challenging to distinguish highly similar cell populations using inter-data evaluation, we next conducted simulations. We designed 20 simulation datasets composed of five cell groups with varying levels of differential expression (DE) scales. Similar to others [22], we used Splatter [23] to pre-define the same set of DE genes in simulation datasets, and only differed the magnitude of DE, from low, low–moderate, moderate to high (Figure 2A). For most methods, the classification task was easier when cell populations were more separable with the higher gene DE scale. As cell populations became less separable with the lower gene DE scale, all methods had a decrease in their performance (Figure 2B) and the degree of such decrease varied among methods. SingleR, RPC, Seurat, SingleCellNet, and CP were in the first class that were relatively more robust than the other five methods. SingleR and RPC were respectively ranked 1st and 2nd for their robustness against cell type similarity, with all three metrics above 0.9. When cells were least separable (low DE), Seurat was ranked 4th after SingleCellNet (which was 3rd), exposing its slight disadvantage. Garnett failed to predict when cell–cell similarity was high (low DE). In this context, the pre-defined marker genes may be ‘ambiguous’ to differentiate multiple cell types, which may cause problems for Garnett to train the classifier.

### The effect of cell type class number on performance

The increased cell type class number imposes a challenge for some methods in intra- and inter-dataset predictions. We designed five simulation datasets, and each was composed of an increased number (*N*) of cell type classes (*N* = 10, 20, 30, 40, 50) with constant total cell number, gene number, and DE level among cell groups. Similar to the performance that we observed on intra-dataset and inter-dataset classification experiments, the increased number of classification labels led to dropping accuracy for most methods, except SingleR, which was extremely robust without drop of performance (Figure 2C). RPC was consistently ranked 2nd regardless of cell type class number. Seurat and CP were respectively ranked 3rd and 4th for their robustness before *N* = 30, with small differences in metrics. However, after *N* = 30, the performance of Seurat deteriorated faster and was ranked 4th. The performance issue in Seurat may be due to its susceptibility towards cell–cell similarity; as we keep a constant DE level despite the increased cell type classes, more cell types have similar expression profiles, and are more likely to be misclassified. On the other hand, Garnett failed to predict when *N* > 20. Therefore, the simulation study confirms the practical challenge of increased cell type classes in the multi-label classification task for most methods. Collectively, results from both real data and simulation data demonstrate that SingleR is the most robust method against increased data complexity.

### The effect of gene filtering on performance stability

**Figure 3:**
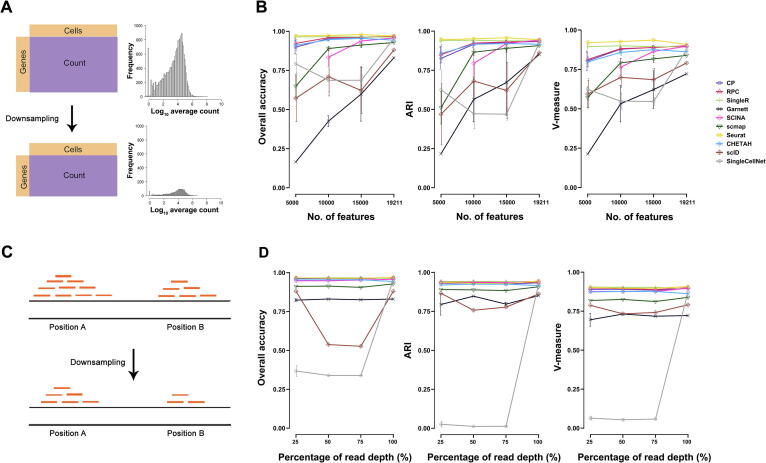
**Effect of gene filtering on annotation performance** **A.** The features (genes) in the human pancreas Fluidigm C1 dataset were filtered by removing genes that are present in less than three cells, resulting in 19,211 genes. The filtered genes were randomly downsampled into 5000, 10,000, and 15,000 input features, following the original log count distribution. Such downsampling was repeated five times. SCINA failed when the number of features reached 5000, thus no point is shown. **B.** Plots depicting three classification metrics (overall accuracy, ARI, and V-measure) of each method applied to downsampling approaches in (A). **C.** The BAM file reads in the human pancreas Fluidigm C1 dataset were randomly downsampled into 25%, 50%, and 75% of the original read depth. **D.** Plots depicting three classification metrics (overall accuracy, ARI, and V-measure) of each method applied to downsampling approaches in (C). In (B) and (D), the *x*-axis is the downsampling size for feature number or read depth, and the *y*-axis is the metric score. Results are shown as mean ± SD. Line colors and point shapes correspond to different methods.

We also evaluated the stability of annotation methods in inter-dataset predictions by downsampling the number of query input features. For this purpose, we used the human pancreas data pair (Table 2). We randomly downsampled the features (genes) from Fluidigm C1 data into 15,000, 10,000 and 5000 input genes, based on the original log count distribution (Figure 3A). When the number of features decreased, most methods had decreased performance metrics as expected (Figure 3B). Seurat and SingleR were the top-two most robust methods over the decreased number of features, and their ARI scores remained high across all sampling sizes (ARI > 0.9). Again, methods such as Garnett, scID, and scmap were more susceptible to low number of features, since their performances decreased as the feature number decreased. Therefore, using query data with fewer features than the reference data may affect the prediction performance of those methods. Alternatively, we also downsized the samples by reducing the number of raw reads before alignment and tag counting steps (Figure 3C). While most methods kept consistent performance metrics with reduced raw reads as expected, a couple of methods, such as SingleCellNet and scID, were perturbed by this procedure (Figure 3D).

### Rare cell type detection

**Figure 4:**
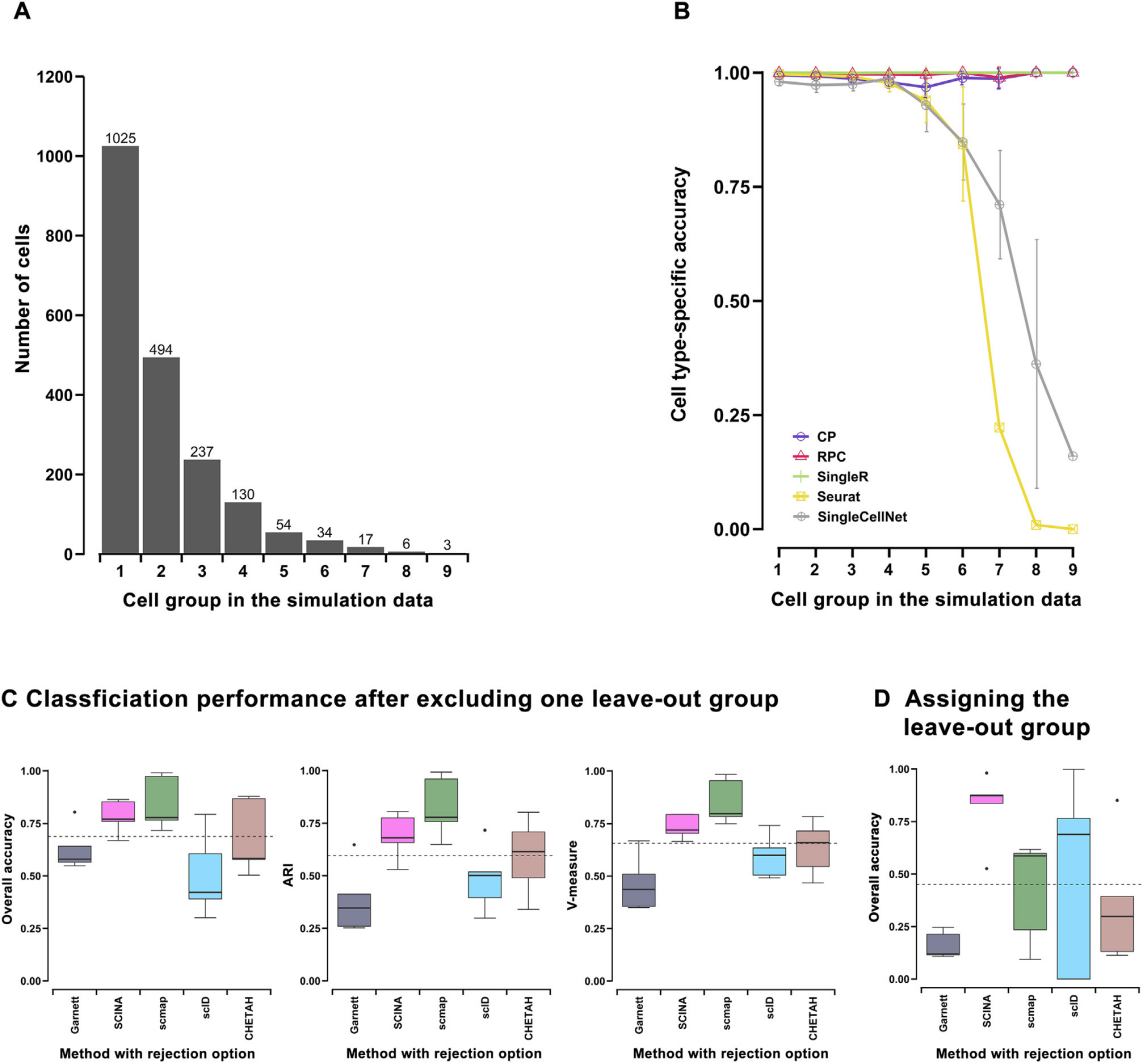
**Performance comparison on rare or unknown cell groupdetection** All datasets were generated by Splatter. **A.** Cell population distribution of simulation data (10 repeats), composed of 10,000 genes and 2000 cells, splitting into 9 cell types with cell number proportions of 51.25%, 24.70%, 11.85%, 6.50%, 2.70%, 1.70%, 0.85%, 0.30%, and 0.15%, respectively. **B.** Plot illustrating cell type-specific accuracy across 9 cell groups in (A), for the five annotation methods with overall accuracy > 0.8 and ARI > 0.8. The *x*-axis is cell groups in descending order for their cell proportions, and the *y*-axis is the cell type-specific accuracy score. Results are shown as mean ± SD over ten repetitions. **C.** Boxplots showing performance metrics (overall accuracy, ARI, and V-measure) of another simulation dataset, composed of 4000 genes and 2000 cells splitting into 5 cell types. During each prediction, one cell group was removed from the reference matrix and the query remained intact. The *x*-axis lists methods with the rejection option (*i.e.*, allowing “unknown” labels), and the *y*-axis is the classification metric after excluding the leave-out group. **D.** A boxplot showing the overall accuracy of methods in (C), when assigning “unknown” class to the leave-out group in the query.

**Supplementary figure S4:**
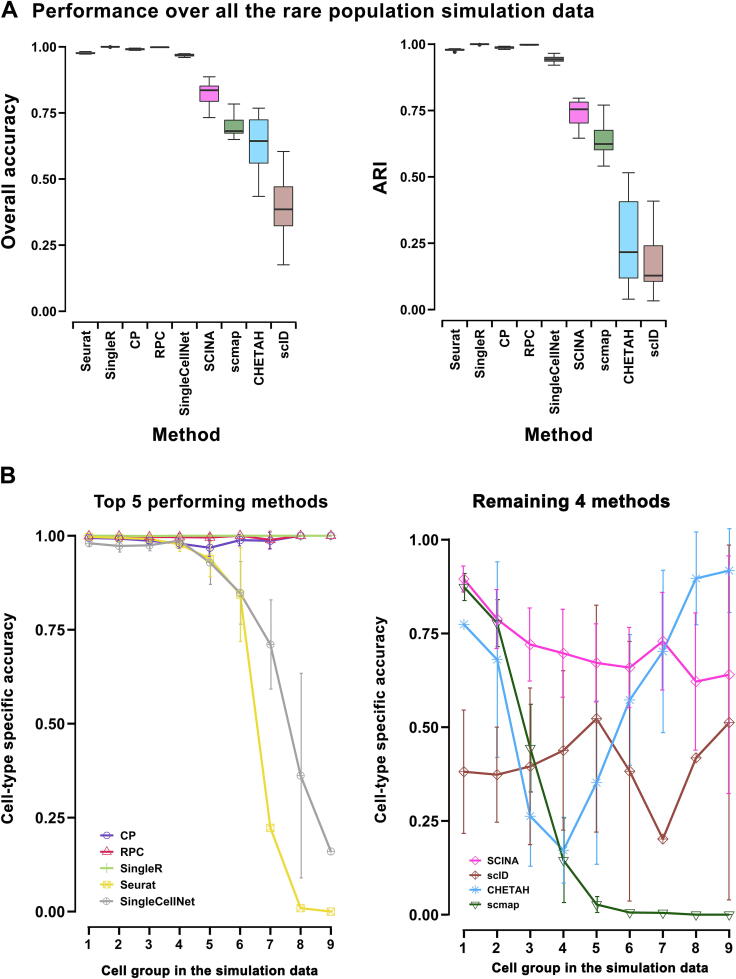
**Rare cell types detection for remaining 4 methods**. **A.** Boxplots illustrating the averaged overall accuracy and ARI over all the rare population detection simulation data. The *x*-axis is the methods evaluated, and the *y*-axis is the metric score. **B.** Rare cell types detection comparing between top 5 performing methods and remaining 4 methods ranked by averaged overall accuracy and ARI. The *x*-axis is the cell groups in descending order for their cell proportions, and the *y*-axis is the cell-type-specific accuracy.

Identifying rare populations in single-cell data is a biologically important aspect. We evaluated the inter-dataset classification accuracy per cell group for the top-five methods selected by overall accuracy and ARI over all the rare population simulation data (Figure S4): Seurat, SingleR, CP, SingleCellNet, and RPC. We used a mixture of nine cell groups with a wide variety of percentages (51.25%, 24.70%, 11.85%, 6.50%, 2.70%, 1.70%, 0.85%, 0.30%, and 0.15%) in ten repeated simulation datasets with different seeds (Figure 4A). When the size of the cell group was larger than 50 cells out of 2000 cells, all five methods achieved high cell type-specific accuracy of over 0.8 (Figure 4B). However, Seurat and SingleCellNet both had a drop of performance when the size of the cell group was 50 cells or less. On the other hand, most low-performing methods had fluctuating performance and did not perform well in classifying major cell groups (Figure S4B). Interestingly, bulk-reference based methods such as SingleR, CP, and RPC were extremely robust against the size change of a cell group as their algorithms use averaged profiles as the reference and are not susceptible to low cell counts. One challenge for some single-cell methods is that there are insufficient cell counts from low-proportion cell types. In fact, some methods remove or ignore low-proportion cell types in the training phase (such as Garnett), or during alignment (such as Seurat) using their threshold parameters.

### Rejection option evaluation

**Supplementary figure S5:**
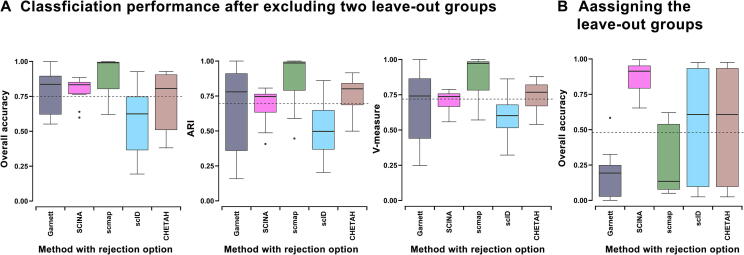
**Leave-out 2-cell-type rejection evaluation**. “Leave-out 2-cell-type” experiment was performed on the same simulation dataset pair used in inter-dataset prediction. In this experiment, signatures of any combination of two cell types were removed in the reference matrix while keeping the query intact. The evaluation was repeated ten times for all ten different combinations. **A.** The *x*-axis lists methods with rejection options (e.g. allowing “unknown” labels), and the *y*-axis is the classification metric after excluding the leave-out groups. **B.** Boxplots showing the accuracy of methods in (A), when assigning “unlabeled” class to leave-out groups in the query.

Among scRNA-seq specific annotation tools, five methods (Garnett, SCINA, scmap, CHETAH, and scID) contain the rejection option that allows “unknown” labels. This is a practical option, as the reference data may not contain all cell labels present in the query data. In order to assess how accurate these methods are at labeling “unknown” cells, we used the scheme of “leave-out 1-cell-type evaluation” on the same simulation dataset pair utilized in the inter-dataset prediction experiment. That is, we removed the signature of one cell type in the reference matrix while keeping the query intact. We repeated the simulation five times for all five cell types. For each method, we measured the classification performance after excluding the leave-out group (Figure 4C), and the overall accuracy of assigning “unknown” to the leave-out group in the query (Figure 4D). Among the five methods compared, SCINA and scmap all had metrics above the average level of all tools tested for performance after excluding the leave-out group (Figure 4C). However, SCINA had a better overall accuracy in rejecting cell types existing in the query dataset but not in the reference (Figure 4D). Similar results were observed from “leave-out 2-cell-type evaluation” (Figure S5). Both evaluations demonstrate that SCINA has a relatively better balance between classification performance in existing cell types and precise rejection of non-existing cell types.

However, the caveat is that none of the rejection-enabled methods are among the best performing methods for evaluations such as inter-dataset predictions and robustness to cell type similarities. Since accuracy, stability, and robustness are probably more important attributes, the ability to detect unknown populations is less useful when the overall accuracy is low.

### Runtime and memory comparison

**Figure 5:**
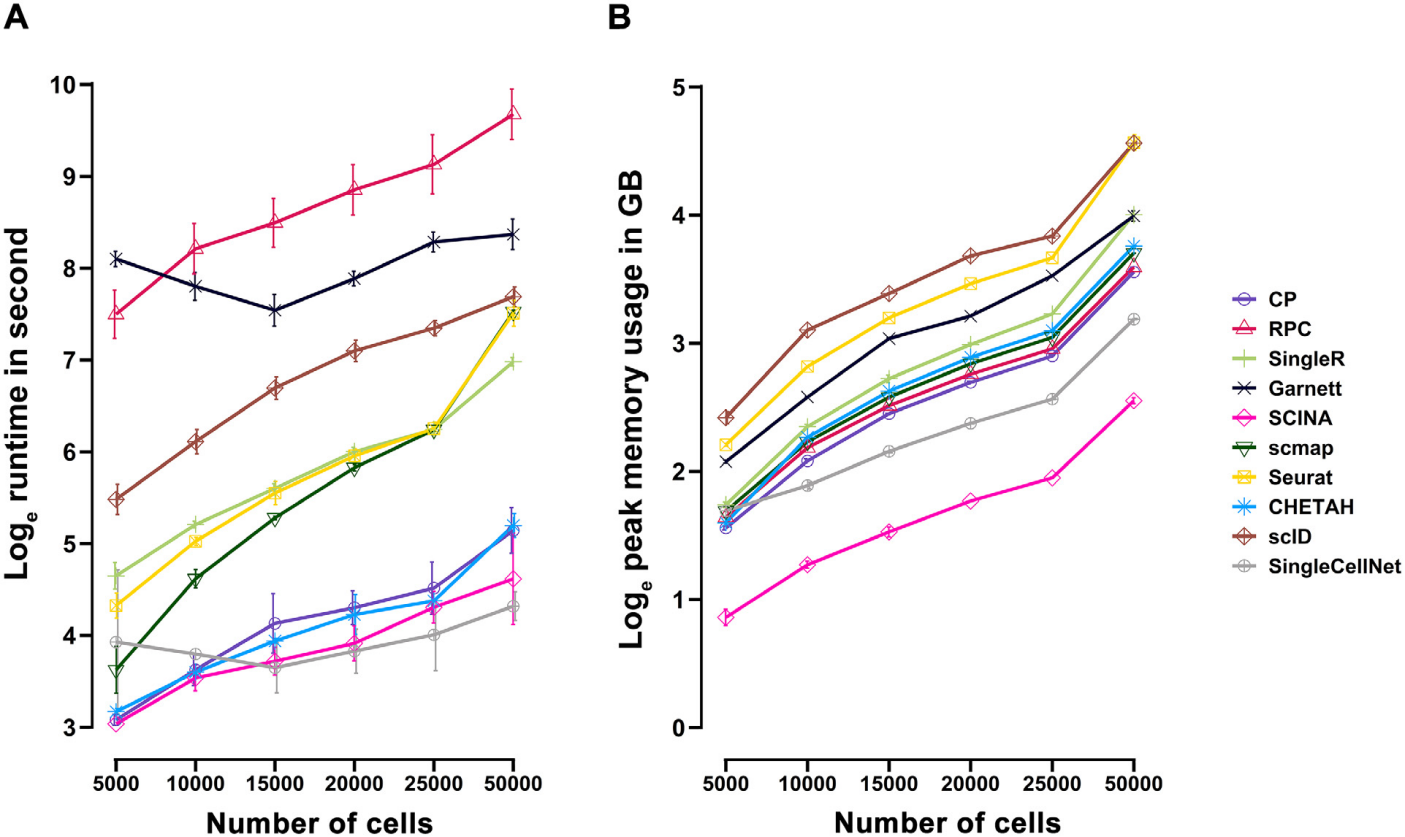
**Speed and memory usage comparison** Speed and memory usage comparison on six pairs of simulation data with increasing number of cells (5000, 10,000, 15,000, 20,000, 25,000, and 50,000). True assay (without dropouts) was used as the reference and the raw assay (with dropout mask) was used as the query. Both reference and query datasets contain the same number of cells. Color depicts different annotation methods. **A.** Natural log of runtime in second (*y*-axis) *vs*. number of cells (*x*-axis) over five repetitions at each data point. **B.** Natural log of peak memory usage in GB (*y*-axis) *vs*. number of cells (*x*-axis) over five repetitions at each data point. Results are shown as mean ± SD. GB, gigabyte.

**Figure 6:**
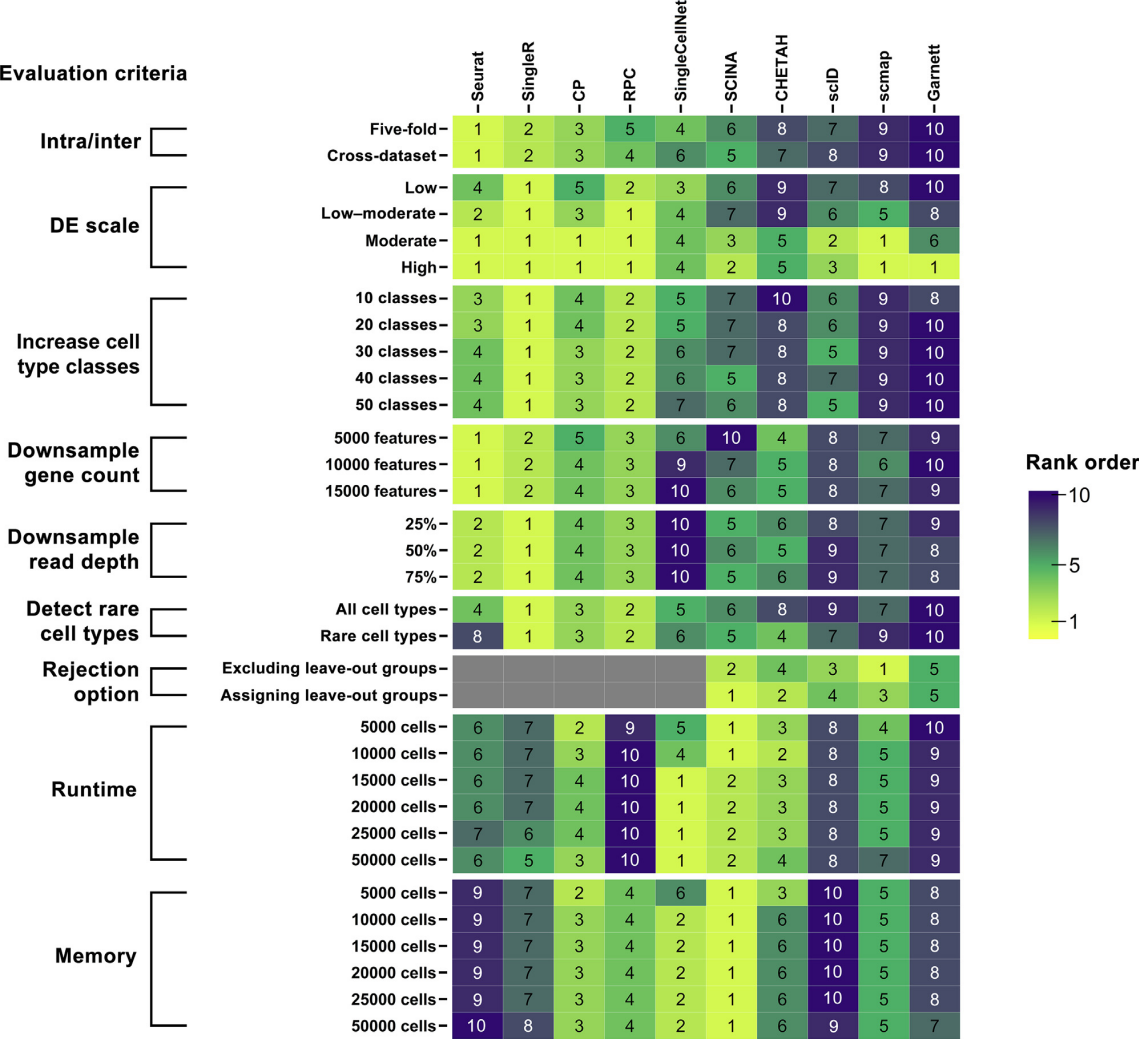
**Benchmark summary** Summary of the classification performance in each evaluation criteria. Each column is a method and each row is an evaluation criterion from intra-dataset and inter-dataset prediction (intra/inter), cell–cell similarity (DE scale), increased cell type classes, downsampling of gene count, downsampling of read depth, rare cell type detection, unknown cell type detection (rejection option), as well as runtime and memory utilization. The heatmap shows the rank of individual methods based on averaged metrics over overall accuracy, ARI, and V-measure for each evaluation indicated in the left row. Rare cell type detection was ranked by averaged cell type-specific accuracy for classifying cell types < 1.70% in population. Unknown cell type detection was ranked by the averaged accuracy of assigning “unknown” to the leave-out group. Runtime and memory were ranked by utilization efficiency. Gray box indicates that the method was not included in the evaluation. The methods in the heatmap are arranged in ascending order by their average rank over intra-dataset and inter-dataset predictions.

In order to compare the runtime and memory utilization of annotation methods, we simulated six pairs of datasets each composed of 20,000 genes, with 5 cell types of equal proportion (20%), in total cell numbers of 5000, 10,000, 15,000, 20,000, 25,000, and 50,000, respectively (see Materials and methods). All methods had increased computation time and memory usage when the number of cells increased (Figure 5). Of the five overall top-performing methods ranked averagely by intra-dataset and inter-dataset annotation evaluations (Seurat, SingleR, CP, RPC, and SingleCellNet) (Figure 6), SingleCellNet and CP outperformed others on speed (Figure 5A). As the dataset size increased beyond 50,000 cells, methods such as RPC required a runtime as long as 6 h. For memory utilization, SingleCellNet and CP consistently required less memory than other three top-performing methods (Figure 5B). Notably, the best performing method Seurat (ranked by intra- and inter-dataset predictions) required memory as large as 100 GB when the data size increased beyond 50,000 cells, which was significantly more than most methods. In all, SingleCellNet and CP outperformed in terms of computational speed and memory efficiency among top-five accurate annotation methods (ranked by intra- and inter-dataset predictions).

## Discussion

In this study, we presented a comprehensive evaluation of ten cell type annotation methods in R packages on scRNA-seq data. Of the ten methods, eight are designed for scRNA-seq data, and two are our unique adaptation from DNA methylation analysis. We evaluated these methods on six publicly available scRNA-seq datasets as well as additional simulation datasets. We systematically assessed the accuracy (through intra-dataset and inter-dataset predictions), the robustness of each method with challenges from gene filtering, cell types with high similarity, and increased cell type classes, the capability on rare or unknown cell type detection, and runtime and memory utilization (Figure 6). In summary, we found that methods such as Seurat, SingleR, CP, RPC, and SingleCellNet performed relatively well overall, with Seurat being the best-performing methods in annotating major cell types. Seurat, SingleR, RPC, and CP were more robust against downsampling of features and read depth. However, Seurat had a major drawback at predicting rare cell types, as well as minor issues at differentiating highly similar cell types and coping with increased cell type classes, compared to SingleR and RPC.

During the manuscript preparation, another evaluation paper was published in a special edition of Genome Biology [24]. We, therefore, address the differences between these two studies’ methodologies and highlight the unique insights of our own, before discussing findings in detail. Firstly, rather than simply comparing methods claimed to be “single-cell specific,” we uniquely repurposed two methods: CP and RPC. Although they were originally developed for DNA methylation data deconvolution, their regression-based principle could be adapted to scRNA-seq supervised or semi-supervised classification. We modified the final regression coefficient as the probability of one specific cell type label, rather than the cell content as in DNA methylation-based deconvolution. As our results indicate, CP and RPC have comparable performance with SingleR (the overall second-best method). This shows the potential of repurposing existing deconvolution methods from another bulk omics analysis. With the rapid accumulation of so-called “single-cell specific” computational methods, where not all are thoroughly evaluated, users (including bioinformaticians) have increasing difficulty in determining which method is most appropriate for a given dataset and biological condition(s). Repurposing pre-existing, sound, and top-performing methods may be an effective alternative approach. Secondly, for benchmark datasets, we used fewer real experimental datasets. However, we uniquely included many simulated datasets while the other study did not use any. We argue that it is important to have additional simulation datasets, as all evaluations based on manually annotated cell type-specific markers in real experimental data are subject to bias. From the computation point of view, simulation datasets can avoid such bias as they provide unambiguous “ground truth,” even under tricky scenarios such as identifying highly similar cell types or very rare cell types. Thirdly, Seurat, the method with the best overall performance in our study, was not included in the other study. The high annotation performance of Seurat on intra-data and inter-data predictions, is mostly due to the fact that it’s a classification method using an integrated reference. Its data transfer feature shares the same anchors’ identification step as the data integration feature. However, unlike data integration, the cell type classification method in Seurat does not correct the query expression data. Additionally, its default setting projects the principal component analysis (PCA) structure of a reference onto the query, instead of learning a joint structure with CCA [10], [21]. Thus, Seurat represents a new kind of “transfer learning” method not discussed in the Genome Biology paper. As the single-cell field develops, more scRNA-seq datasets of similar conditions need comparative analysis, and more multi-omics data will be generated from the same single cells. Forward-thinking methods, such as Seurat and a series of scRNA-seq data integration methods (LIGER, Harmony, scAlign, *etc.* [25], [26], [27]) will allow seamless data integration, and automatic cell annotation will be part of this process. Lastly, we only selected packages in R with good documentation, as R is still the most popular bioinformatics platform for open-source scRNA-seq analysis packages.

Although having slightly lower performance metrics than Seurat, SingleR and CP still have excellent intra-data and inter-data prediction performances, with resilience toward gene filtering and increased complexity in datasets. In addition, SingleR has a better performance than Seurat in predicting rare cell types, dealing with increased cell type classes, and differentiating highly similar cell types. This advantage of SingleR may benefit from its method and the pseudo-bulk reference matrix. SingleR uses pseudo-bulk RNA-seq reference to correlate averaged cell type expression profiles to each of the single cells in query data, and it uses highly variable genes to find the best fit iteratively. The averaged pseudo-bulk reference profile may potentially remove the variation and noise from the original single-cell reference profile. In addition, it can retain expression profiles of all cell types and is not affected by the low cell count for rare cell types. For Seurat, the annotation of cell labels on query data is informed by the nearest anchor pairs. If two or more cell types have similar profiles, their alignments may overlap, which could cause misclassification. Seurat also has requirements on the minimum number of defined anchor pairs. In the case of rare cell types, the lack of the neighborhood information makes the prediction difficult. Similar to other study [24], we also found that the method that incorporates the prior knowledge (*e.g.*, Garnett and SCINA) does not improve the classification performance over other methods without such requirements. This prior knowledge is limited when cell–cell similarity is large. In addition, as the number of cell types increases, the search for marker genes will become more challenging, making these methods even less desirable.

Compared with intra-dataset prediction, inter-dataset prediction is more realistic but also more challenging. Technical, platform, and batch differences in inter-dataset prediction may impose major challenges to the classification process, even when tissue and cell type contents are the same. In our study, the CCA batch-correction preprocessing step did not improve the classification accuracy for most methods. Among all experimental data used as the benchmark in this study, PBMC datasets had the worst accuracy results (ARI = 0.76 for the best method Seurat). Further inspection of the confusion matrix revealed that the challenge came from distinguishing highly similar cell types, which themselves may have some level of inaccuracy from the original experiments. If the upstream unsupervised clustering methods are not sufficiently sensitive to categorize similar cell populations, this uncertainty may be carried through to the downstream cell annotation step. This again highlights the potential issue of evaluating supervised or semi-supervised methods in single-cell data, where we are not certain about the “ground truth” of cell labels to begin with. Recently, some studies improved unsupervised clustering methods through multi-omics integration, and/or reconstruction of gene regulatory networks [28], [29], representing a new trend in this area. As the multi-omics technology continues to advance [30], where both multi-omics and pre-defined marker information are available for the same sample, we can expect both unsupervised clustering methods and cell type annotation methods to be further improved.

Overall, we recommend using Seurat for general annotation tasks for cell types that are relatively separable and without rare cell type identification as the objective. However, for datasets containing cell types with high similarities or rare cell populations, if a reference dataset with clean annotations is available, SingleR, RPC, and CP are preferable.

## Materials and methods

### Real datasets

Six real scRNA-seq datasets were downloaded and used for evaluations and validations (Table 2). Two human pancreatic islet datasets [10], [31], [32] were obtained via Gene Expression Omnibus (GEO: GSE85241 and GSE86469; https://www.ncbi.nlm.nih.gov/geo/). The *Tabula Muris* datasets Version 2 [3] were downloaded from the Chan Zuckerberg Biohub (https://tabula-muris.ds.czbiohub.org/). The bead-purified PBMC dataset [33] was obtained from the Zheng dataset (https://github.com/10XGenomics/single-cell-3prime-paper), and the PBMC-3K dataset was downloaded from 10X Genomics (https://support.10xgenomics.com/single-cell-gene-expression/datasets/).

These datasets differ by species, tissue contents, and sequencing protocols. For each dataset, we collected both the raw count matrix and cell type annotations from the corresponding publication, except PBMC-3K, for which cell type annotations were obtained through the standard scRNA-seq analysis and classified using cell type-specific marker genes. The extracted cell type annotations for each dataset were used as the ground truth for evaluations (Table S1).

### *Data cleaning*

Datasets were paired in groups by tissue types (Table 2). Within a pair, we used the data generated by the FACS method as reference data. Both reference data and query data were further processed to ensure the number of cell types in reference data was larger or equal to that in query data. When necessary, query data were downsampled following the original cell type count distribution. For the two TM full datasets from 10X and Smart-Seq2 platforms, which contain a large number of cell types (32 and 37 cell types, respectively), we took a subset to create two TM lung datasets (8 and 10 cell types, respectively). As a result, we generated four pairs of experimental datasets: PBMC pair with PBMC sorted data as the reference and PBMC-3K data as the query; human pancreas cell pair with pancreas CEL-Seq2 data as the reference and pancreas Fluidigm C1 data as the query; TM full pair with TM full Smart-Seq2 data as the reference and TM full 10X data as the query; TM lung pair with TM lung Smart-Seq2 data as the reference and TM lung 10X data as the query.

### *Data downsampling*

To explore the effects of different feature number and read depth on the performance of tools, we randomly downsampled features (genes) from human pancreas Fluidigm C1 dataset into 5000, 10,000 and 15,000 input genes, following the original log count distribution. We repeated five times for each downsampling scheme. We also downsampled the reads into 25%, 50%, 75% of the original read depth (with two repetitions) on BAM files, and then realigned files following the method provided by the original manuscript [32].

### Simulated datasets

We first simulated a dataset using Splatter, with 4000 genes and 2000 cells (Splatter parameters, dropout.shape = –0.5, dropout.mid = 1), and then split the dataset into five cell groups with proportions of 10%, 30%, 30%, 10%, and 20%. In addition, we also generated four additional simulation sets to evaluate the robustness of tools. The first set contains 20 simulation datasets, with each composed of 10,000 genes and 2000 cells splitting into 5 cell types with equal proportion. These datasets have the same set of DE genes but differ by the magnitude of DE factors. We simulated each DE scale five times with five different seeds. The DE scale and the parameterization in Splatter were: low: de.facScale = (0.1, 0.3, 0.1, 0.3, 0.2); low–moderate: de.facScale = (0.3, 0.5, 0.3, 0.5, 0.4); moderate: de.facScale = (0.5, 0.7, 0.5, 0.7, 0.6); high: de.facScale = (0.7, 0.9, 0.7, 0.9, 0.8). The second set contains five simulation datasets each composed of an increased number (*N*) of cell type classes (*N* = 10, 20, 30, 40, 50) with constant total cell number (10,000), gene number (20,000), and DE level among cell groups. In the third set, we generated ten simulation datasets each with 10,000 genes and 2000 cells (using ten different seeds), and then split each into 9 cell groups with proportions of  51.25%, 24.70%, 11.85%, 6.50%, 2.70%, 1.70%, 0.85%, 0.30%, and 0.15%, respectively. The fourth simulation set contains six datasets with total cell numbers of 5000, 10,000, 15,000, 20,000, 25,000, and 50,000, respectively. Each dataset contains 20,000 genes and 5 cell types with the equal proportion.

Each simulation dataset contains two paired assays. The true assay without dropouts was used as the reference and the raw assay with dropout mask was used as the query.

### Data preprocessing

#### Cell and gene filtering

We filtered out cells for which fewer than 200 genes were detected and genes that were expressed in fewer than 3 cells.

#### Normalization

For annotation tools that require a normalized count matrix as the input, we performed log-normalization using a size factor of 10,000.

#### Pseudo-bulk reference matrix

For annotation tools that use bulk rather than single-cell expression profiles as reference, we took the average of the normalized count of each cell type group and made a pseudo-bulk RNA-seq reference.

#### Marker gene selection

Some classification tools (SCINA and Garnett) require cell type-specific markers as the input. When such marker information was neither provided by the corresponding tools nor retrievable by public research, we extracted them from the reference data by performing differential expression analysis using Wilcoxon rank sum test (FindAllMarkers function from Seurat with parameters only.pos = TRUE, min.pct = 0.25, and logfc.threshold = 0.25). Wilcoxon rank sum test is the most common nonparametric test for a difference in mean expression between cell groups. The ten top-ranked marker genes for each cell type were used as the input for corresponding tools.

### Annotation methods

We only considered pre-printed or published methods with detailed documentation on installation and execution. We excluded any methods that required extensive runtime, and where we were unable to customize the reference dataset, or inconsistent predictions were produced. In the end, ten cell annotation methods, publicly available as R packages, were evaluated in this study. This included eight commonly used scRNA-seq annotation methods: Seurat, scmap, SingleR, CHETAH, SingleCellNet, scID, Garnett, and SCINA. In addition, to investigate the potential to repurpose deconvolution methods for other bulk omics analysis, we also included and modified two methods originally designed for DNA methylation analysis that use algorithms not yet reported in scRNA-seq specific tools: CP and RPC.

All parameters were set to default values following the authors’ recommendations or the respective manuals (Table 1). For methods that allow “unknown” assignments (scmap, CHETAH, scID, Garnett, and SCINA), we modified the parameter to force assignments where possible (except for evaluations where unknown assignments were allowed).

#### Adaptation of CP and RPC methods for scRNA-seq analysis

In order to accommodate the methylation-based methods for scRNA-seq data, we made some modifications. In original papers, both RPC and CP model the methylation profile of any given sample as a linear combination of a given reference profile representing underlying cell types present in the sample. We assumed that the number of underlying cell types to be *C*, and each cell type has a profile *b_c_* that constitutes the signature matrix H [34], [35], [36]. Let ***y*** be the profile of a given sample and *w_c_* be the weight estimation of cellular proportion of each cell type, and the underlying model becomes:

$$y=\overset{C}{\underset{c=1}{\sum }}w_{c}b_{c}+∊$$

Both methods assume that reference profiles contain the major cell types present in the sample ***y*** and sum of weights equal to 1. RPC estimates the weight coefficient using robust multivariate linear regression or robust partial correlation, while CP uses a quadratic programming technique known as linear constrained projection to estimate the weights [37].

**Supplementary figure S6:**
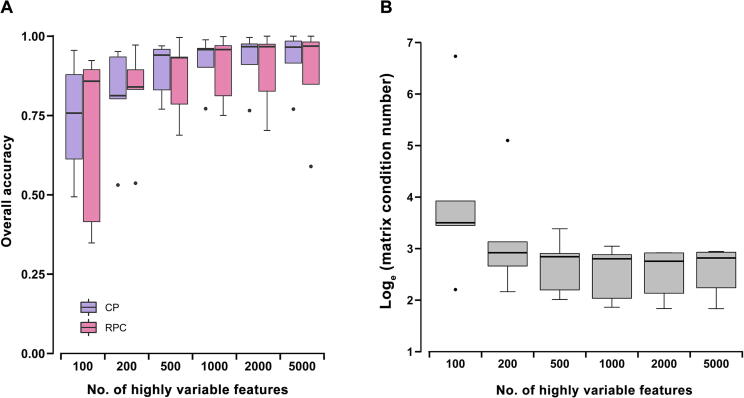
**The optimal number of highly variable genes to be used in CP and RPC algorithms**. The highly variable genes were identified from the reference dataset and ranked by standardized variance from the mean-variance feature selection method with variance-stabilizing transformation. **A.** The boxplot depicts the overall accuracy averaged over inter-dataset predictions (PBMC, pancreas, TM full, TM lung, and simulation) with the top 100, 200, 500, 1000, 2000, and 5000 highly variable genes as input features for CP and RPC methods. The *x*-axis is the number of highly variable features, and the *y*-axis is the overall accuracy. Methods are reflected by different box colors. **B.** The boxplot represents the condition number of the pseudo-bulk reference matrix averaged over inter-dataset predictions with the top 100, 200, 500, 1000, 2000, and 5000 highly variable genes as input features. The *x*-axis is the number of highly variable features, and the *y*-axis is the condition number.

In the modified version, we first converted the scRNA-seq reference data into pseudo-bulk RNA-seq data matrix by taking the average of the normalized count of each cell type group. Then we took a subset of pseudo-bulk RNA-seq matrix with a small condition number by keeping 2000 features that exhibited high cell-to-cell variations across *C* distinct cell types, as the signature matrix H [34]. We set the highly variable genes to 2000, using FindVaraibleFeatures function from Seurat (Figure S6). We let *y* be the profile of a given single cell from the query data with the same 2000 genes from the signature matrix H. While applying both algorithms, we treated the estimated weight for each cell type as the probability and the cell type with the highest weight was the identity of the corresponding single-cell sample in the query data. This conversion is based on the fact that *y* no longer represents averages over many different cell types, but instead contains expression profiles from one cell type (since we have single-cell data).

### Benchmarking

#### Five-fold cross validation and inter-dataset prediction

For each dataset in four pairs of real experimental datasets mentioned above, we used a 5-fold cross validation where the 4-fold data were used as the reference and the remaining 1-fold as the query. For the inter-dataset prediction, in addition to the four pairs of real datasets, we used a pair of simulation datasets containing true assay (without dropouts) as the reference and raw assay (with dropout mask) as the query.

To evaluate whether batch correction and data integration benefit the classification performance, for each pair of real datasets, we aligned both reference and query data using CCA [10], [21] from Seurat data integration function. Then we separated the aligned data and performed the inter-dataset evaluation again.

#### Performance evaluation on the effect of feature number and read depth

To investigate the robustness of different methods with regards to feature number and read depth, we used the downsampled human pancreas Fluidigm C1 dataset as described in the data downsampling section. In such evaluation, the human pancreas CEL-Seq2 dataset was used as the reference and the downsampled human pancreas Fluidigm C1 dataset was used as a query.

#### Performance evaluation on the effect of DE scale

In this assessment, we used 20 simulation datasets containing the same DE gene set but differing only by DE factors as described earlier in the simulated datasets section. Each simulation dataset contains two paired assays. The true assay (without dropouts) was used as the reference and the raw assay (with dropout mask) was used as the query.

#### Performance evaluation on the effect of classification labels

In this evaluation, we designed five simulation datasets, each composed of an increased number (*N*) of cell groups (*N* = 10, 20, 30, 40, 50) with constant total cell number, gene number, and DE level among cell groups. Each simulation dataset contains two paired assays. The true assay (without dropouts) was used as the reference and the raw assay (with dropout mask) was used as the query.

#### Rare and unknown cell type detection

Each of the ten simulation datasets in the rare cell type detection was composed of 10,000 genes and 2000 cells splitting into 9 cell types with proportions of 51.25%, 24.70%, 11.85%, 6.50%, 2.70%, 1.70%, 0.85%, 0.30%, and 0.15%, respectively. The simulation dataset in the unknown cell type detection was composed of 4000 genes and 2000 cells splitting into 5 cell types. We used the scheme of “leave-out 1-cell-type evaluation” to evaluate prediction on the unknown cell groups, that is, removing the signature of one cell type in the reference matrix while predicting the query. During each prediction, one cell group was removed from the reference matrix and the query remained intact. We repeated the evaluation five times for all five cell types. We additionally employed a “leave-out 2-cell-type evaluation”, in which we removed signatures of any combination of two cell types in the reference matrix while keeping the query intact. The evaluation was repeated ten times for all ten different combinations. Similarly, for each simulation dataset, the true assay (without dropouts) was used as the reference and the raw assay (with dropout mask) was used as the query.

#### Runtime and memory *utilization* assessment

To compare the computational runtime and memory utilization of annotation methods, we simulated six datasets, with total cell numbers of 5000, 10,000, 15,000, 20,000, 25,000, and 50,000, respectively. Each dataset contains 20,000 genes and 5 cell types with equal proportion. The true assay (without dropouts) was used as the reference, and the raw assay (with dropout mask) was used as the query. Each execution was performed in a separate R session on our lab server [four nodes (Dell PowerEdge C6420) of 2X Intel(R) Xeon(R) Gold 6154 CPU @ 3.00 GHz, 192 GB RAM, one node (Dell Poweredge R740) with 2X Xeon(R) Gold 6148 CPU @ 2.40 GHz, 192 GB RAM, and two 16 GB Nvidia V100 GPUs] with Slurm job scheduler. One processor and 100 GB memory were reserved for each job. From the job summary, we collected ‘Job Wall-clock time’ and ‘Memory Utilized’ for evaluation. We ran each method on each dataset five times to estimate the average computation time and memory usage.

### Evaluation criteria

The prediction results of all methods were evaluated using three different metrics: overall accuracy, ARI, and V-measure. We used three different metrics to avoid possible bias in evaluating the performance. The detailed explanations on these metrics were described earlier [22], [38], [39]. Briefly, overall accuracy is the percent agreement between the predicted label and the true label. ARI is the ratio of all cell pairs that are either correctly classified together or correctly not classified together, among all possible pairs, with adjustment for chance. V-measure is the harmonic mean of distinct homogeneity and completeness score. In specific, homogeneity was used to assess whether each predicted cell type group contains only members of a single class, while completeness was used to assess whether all members of a given class are assigned to the same predicted cell label.

## Availability

All the code and data are available from https://github.com/qianhuiSenn/scRNA_cell_deconv_benchmark.

## CRediT author statement

**Qianhui Huang:** Data curation, Formal analysis, Methodology, Writing - original draft, Writing - review & editing. **Yu Liu:** Methodology, Writing - review & editing. **Yuheng Du:** Validation, Writing - review & editing. **Lana X. Garmire:** Conceptualization, Supervision, Writing - review & editing. All authors read and approved the final manuscript.

## Competing interests

The authors have declared no competing financial interests.

## Supplementary material

Supplementary Table S1 **Composition of cell-types in each real dataset**.
