# Supplementary material for: Evaluation of Cell Type Annotation R Packages on Single-cell RNA-seq Data
Source: Genomics Proteomics Bioinformatics. 2020 Dec 24;19(2):267–81. doi: 10.1016/j.gpb.2020.07.004 (PMC8602772; doi:10.1016/j.gpb.2020.07.004)
Supplement: Supplementary Table S1 — Composition of cell-types in each real dataset. [file mmc1.docx]

**Table S1 Composition of cell-types in each real dataset**

| **PBMC-Sorted** | **PBMC-3K** | **Pancreas Cel-seq2** | **Pancreas-Fluidigm** | **TM-lung Smart-seq2** | **TM-lung 10X** | **TM-Full Smart-seq2** | **TM-Full 10X** |
| --- | --- | --- | --- | --- | --- | --- | --- |
| B cell (10,084) | B cell (342) | Acinar (274) | Acinar (21) | B cell (57) | B cell (140) | B cell (2029) | B cell (5615) |
| CD14^+^ monocyte (2465) | CD14^+^ monocyte (2465) | Activated stella (90) | Activated stella (16) | Ciliated columnar cell (25) | - | Basal cell (1340) | Basal cell (27) |
| CD34^+^ (6312) | - | Alpha (843) | Alpha (239) | Classical monocyte (90) | Classical monocyte (4) | Basal cell of epidermis (1648) | Basal cell of epidermis (2020) |
| CD4^+^ T cell (42,166) | CD4^+^ T cell (479) | Beta (445) | Beta (258) | Leukocyte (35) | Leukocyte (9) | Basophil (25) | - |
| CD8^+^ T cell (22,138) | CD8^+^ T cell (308) | Delta (203) | Delta (25) | Lung endothelia cell (693) | Lung endothelia cell (24) | Bladder cell (695) | Bladder cell (192) |
| Dendritic cell (99) | Dendritic cell (33) | Ductal (258) | Ductal (36) | Monocyte (65) | - | Bladder urothelial cell (683) | Bladder urothelial cell (141) |
| NK cell (8385) | NK cell (155) | Endothelial (21) | Endothelial (14) | Myeloid cell (85) | Myeloid cell (2) | Blood cell (206) | Blood cell (153) |
| - | - | Epsilon (4) | Epsilon (1) | NK cell (37) | NK cell (113) | Cardiac muscle cell (133) | - |
| - | - | Gamma (110) | Gamma  (18) | Stromal cell (423) | Stromal cell (888) | Ciliated columnar cell (25) | - |
| - | - | Macrophage (15) | Macrophage (1) | T cell (53) | T cell (123) | Classical monocyte (90) | Classical monocyte (4) |
| - | - | Mast (6) | Mast (3) | - | - | DN1 thymic pro-T cell (32) | - |
| - | - | Quiescent stella (12) | Quiescent stella (1) | - | - | Endocardial cell (165) | Endocardial cell (1) |
| - | - | Schwann (4) | Schwann (5) | - | - | Endothelial cell (3319) | Endothelial cell (971) |
| - | - | - | - | - | - | Endothelial cell of hepatic sinusoid (182) | - |
| - | - | - | - | - | - | Epithelial cell (201) | Epithelial cell (99) |
| - | - | - | - | - | - | Fibroblast (2189) | Fibroblast (4) |
| - | - | - | - | - | - | Granulocyte (761) | Granulocyte (73) |
| - | - | - | - | - | - | Granulocytopoietic cell (221) | Granulocytopoietic cell (14) |
| - | - | - | - | - | - | Hematopoietic precursor cell (265) | Hematopoietic precursor cell (24) |
| - | - | - | - | - | - | Hepatocyte (391) | Hepatocyte (374) |
| - | - | - | - | - | - | Immature B cell (344) | Immature B cell (3) |
| - | - | - | - | - | - | Immature T cell (1337) | Immature T cell (222) |
| - | - | - | - | - | - | Keratinocyte (330) | Keratinocyte (1035) |
| - | - | - | - | - | - | Kidney collecting duct epithelial cell (121) | Kidney collecting duct epithelial cell (24) |
| - | - | - | - | - | - | Late pro-B cell (306) | Late pro-B cell (14) |
| - | - | - | - | - | - | Leukocyte (683) | Leukocyte (19) |
| - | - | - | - | - | - | Luminal epithelial cell of mammary gland (578) | Luminal epithelial cell of mammary gland (35) |
| - | - | - | - | - | - | Lung endothelial cell (693) | Lung endothelial cell (24) |
| - | - | - | - | - | - | Macrophage (395) | Macrophage (208) |
| - | - | - | - | - | - | Mesenchymal cell (830) | Mesenchymal cell (5200) |
| - | - | - | - | - | - | Mesenchymal stem cell (499) | Mesenchymal stem cell (169) |
| - | - | - | - | - | - | Monocyte (331) | Monocyte (42) |
| - | - | - | - | - | - | Myeloid cell (1208) | Myeloid cell (2) |
| - | - | - | - | - | - | NK cell (171) | NK cell (142) |
| - | - | - | - | - | - | Skeletal muscle satellite cell (540) | Skeletal muscle satellite cell (11) |
| - | - | - | - | - | - | Stromal cell (863) | Stromal cell (1153) |
| - | - | - | - | - | - | T cell (793) | T cell (1985) |

*Note*: Values are indicated as cell type (cell count). TM, Tabula Muris.
